# Adolescent Epstein–Barr virus–associated cholestatic jaundice with porta hepatis/pancreatic head lymphadenopathy and transient CA 19-9 elevation: a case report

**DOI:** 10.3389/fped.2026.1760882

**Published:** 2026-03-16

**Authors:** Ying Chen, Bo Liu, Xiaoyu Zhu

**Affiliations:** 1 Department of Gastroenterology, Yibin Hospital Affiliated to Children's Hospital of Chongqing Medical University, Yibin, China; 2 Department of Gastroenterology, National Clinical Research Center for Child Health and Disorders, Ministry of Education Key Laboratory of Child Development and Disorders, Children's Hospital of Chongqing Medical University, Chongqing, China

**Keywords:** adolescent, CA 19-9, cholestatic jaundice, Epstein–Barr virus, infectious mononucleosis, lymphadenopathy, MRCP

## Abstract

**Background:**

Cholestatic jaundice is uncommon in adolescents. Epstein–Barr virus (EBV)–related inflammatory lymphadenopathy can mimic malignant biliary obstruction and cause a transient rise in carbohydrate antigen 19-9 (CA 19-9), complicating diagnosis.

**Case presentation:**

A previously healthy 15-year-old girl presented with 6 days of jaundice and dark urine without fever. Physical examination showed moderate generalized jaundice with mild scleral icterus; multiple mobile, non-tender lymph nodes in the bilateral cervical regions and right post-auricular area (largest ∼2 × 2 cm); palpable liver and spleen; and mild right upper-quadrant tenderness with equivocal Murphy's sign. Initial tests showed a cholestatic pattern (total/direct bilirubin 102.4/67.7 μmol/L at the referring hospital; and 133.2/126.3 μmol/L on admission) with elevated ALP/GGT and total bile acids. CT suggested a porta hepatis-to-pancreatic-head soft-tissue density with mild intrahepatic ductal prominence; CA 19-9 was 117.7 U/mL [ref <37 U/mL]. EBV POCT was positive; EBV DNA was detected at low level. MRI/MRCP (2025-09-29) showed no obvious biliary obstruction. After MRCP excluded fixed biliary obstruction, the patient improved with conservative management (diagnostic evaluation, close monitoring, and symptom-directed care) without invasive intervention; CA 19-9 declined. No causal inference regarding any medication effect should be drawn, and we do not recommend self-medication or routine off-label/unlicensed use of cholestasis-directed agents for EBV infection. In immunocompetent hosts, EBV-related lymphadenopathy is usually self-limited, and this case does not represent EBV-associated lymphoproliferative disease.

**Conclusion:**

EBV-related cholestatic hepatitis in adolescents can present with transient CA 19-9 elevation and reactive porta hepatis/pancreatic head lymphadenopathy, mimicking malignant obstruction. Serial laboratory and imaging assessments—particularly MRCP—together with conservative management may obviate unnecessary invasive procedures.

## Introduction

The differential diagnosis of cholestatic jaundice in children and adolescents includes mechanical obstruction (stones, strictures, tumors), inflammatory edema, immune-mediated cholangiopathies, drug-induced liver injury, and infections (3). EBV typically presents with fever, pharyngitis, and lymphadenopathy; cholestasis with obstructive-appearing jaundice is less common (4–12). Although CA 19-9 is often regarded as a marker for pancreatobiliary malignancies, benign cholestasis can also increase CA 19-9 (5–15). Distinguishing reversible inflammatory processes from malignancy is crucial to avoid overtreatment (6, 7, 16). We report an adolescent case characterized by cholestatic jaundice, porta hepatis/pancreatic head lymphadenopathy, EBV positivity, and moderate CA 19-9 elevation, with improvement under conservative therapy. This case report was prepared in accordance with the CARE guidelines (1, 2).

**Figure 1:**
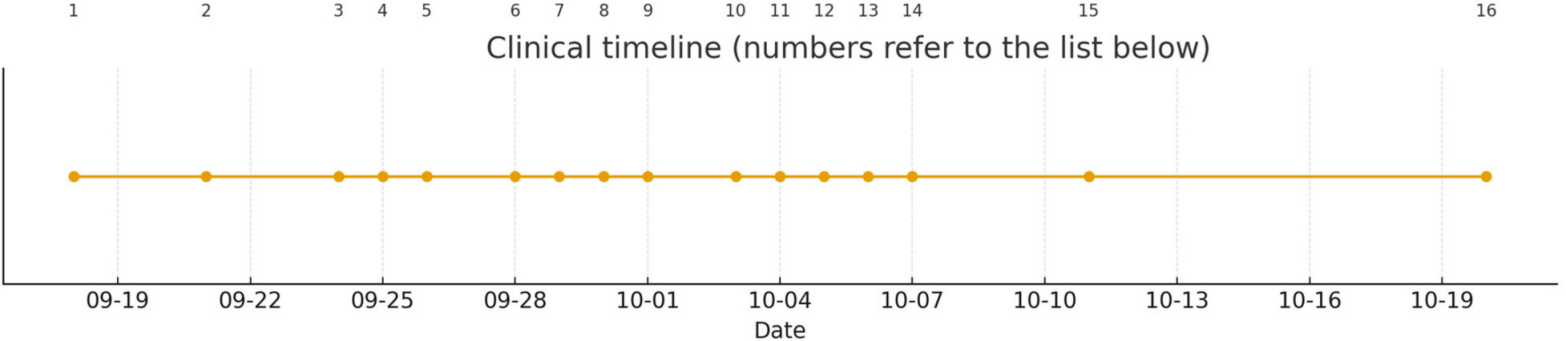
Clinical timeline (see Table 2 for a structured correlation of time points with laboratories, imaging, and clinical interpretation).

## Case presentation

### Patient information

We report the case of a 15-year-old girl of Han ethnicity from Sichuan, China, who presented with progressive jaundice and dark urine for approximately 6 days prior to admission (symptom onset: 2025-09-18) without fever. Three days later, she developed mild scleral icterus, dizziness, fatigue, and reduced appetite; on the day of admission she noted mild right upper-quadrant discomfort. She denied pruritus, acholic stool, rash, bleeding, or neuro-respiratory symptoms. She had no known drug or food allergies and was not taking any regular medications. Menarche occurred at 13 years with irregular cycles. Her parents were non-consanguineous, and there was no family history of hereditary disease. Vaccinations were reported to be up to date.

### Physical examination at admission (2025-09-24)

T 36.3 °C, HR 104/min (regular), RR 19/min (smooth), BP 94/67 mmHg; weight 63.5 kg; height 180 cm. According to the WHO 2007 growth reference for girls aged 5–19 years, her height (180 cm) corresponded to approximately the 99.6th percentile (z = +2.66), and BMI (19.6 kg/m^2^) to the 41st percentile (z = −0.23). WHO does not recommend weight-for-age percentiles beyond 10 years; therefore, weight is reported together with BMI-for-age. General condition: Conscious, alert, nutrition good, no acute distress. Skin and mucous membranes: Moderate generalized jaundice (sclera, face, trunk, and extremities were icteric; scleral icterus was pale lemon-colored); no skin pruritus scratches, no rashes, no petechiae, no spider nevi, no palmar erythema. Lymph nodes: Multiple lymph nodes were palpable in bilateral cervical regions (levels I–III) and right post-auricular area; the largest was approximately 2 × 2 cm, with moderate texture, good mobility, no tenderness, no fusion, and no ulceration. Head, eyes, ears, nose, throat: Conjunctivae not congested; tympanic membranes intact; pharynx not hyperemic; tonsils not enlarged. Chest: Respiratory sounds clear bilaterally, no rales or rhonchi. Heart: Heart rate regular, no cardiac murmurs. Abdomen: Abdomen flat and soft, no abdominal wall varices. Liver edge was palpable 2 cm below the right costal margin at the midclavicular line, smooth, non-nodular, and non-tender; the upper border of hepatic dullness on percussion was not displaced. There was no clinically appreciable hepatomegaly beyond this finding, and no ascites. Hepatic percussion tenderness was negative. Spleen was palpable 1 cm below the left costal margin, soft in texture, non-tender, and smooth on surface. Mild tenderness in the right upper quadrant (patient complained of slight discomfort during palpation, no obvious evasion); Murphy's sign was equivocal (no significant aggravation of tenderness in the right upper quadrant during deep inspiration, no breath-holding movement). No rebound tenderness or muscle guarding; shifting dullness was negative; bowel sounds were normal (4–5 times per minute), no vascular murmurs. Extremities and nervous system: No edema of extremities; muscle strength and tone normal; neurological reflexes intact. Back examination showed no costovertebral angle tenderness. Apart from the cervical/post-auricular nodes described above, no axillary or inguinal lymphadenopathy was detected. External genital examination was unremarkable. Apart from mild splenomegaly and cervical/post-auricular lymphadenopathy, no other organomegaly was identified.

### Investigations

Investigations outside hospital were as follows:

Outside hospital (2025-09-21): WBC 14.37 × 10⁹/L [ref 4.0–10.0 × 10⁹/L] (lymphocytes 75.3% [ref 20%–40%]); Hb 134 g/L [ref 120–160 g/L]; PLT 140 × 10⁹/L [ref 150–400 × 10⁹/L]. Total/direct bilirubin 102.4/67.7 μmol/L [ref 3.4–20.5 μmol/L/0–6.8 μmol/L]; ALT/AST 310.1/157.1 U/L [ref 7–40 U/L/13–35 U/L]; ALP/GGT 253.5/243.3 U/L [ref 45–150 U/L (age-dependent)/7–45 U/L]; total bile acids 138.5 μmol/L [ref 0–10 μmol/L]. Urine bilirubin 3+, urobilinogen 3+. IgM antibodies against Hepatitis A–E were negative and thyroid function tests were normal. Direct antiglobulin test positive. Ultrasound: small gallbladder with poor filling; possible wall thickening.

At admission (2025-09-24): WBC 17.00 × 10⁹/L [ref 4.0–10.0 × 10⁹/L] (lymphocytes 84.6% [ref 20%–40%], neutrophils 12.2% [ref 40%–75%]); PLT 133 × 10⁹/L [ref 150–400 × 10⁹/L]; Hb 128 g/L [ref 120–160 g/L]; reticulocytes 0.095 × 10^12^/L [ref 0.02–0.10 × 10^12^/L] (2.16% [ref 0.5–2.5%]); SAA 16.91 mg/L [ref <10 mg/L]; lactate 2.67 mmol/L [ref 0.5–2.2 mmol/L]. Liver tests: total/direct bilirubin 133.2/126.3 μmol/L [ref 3.4–20.5 μmol/L/0–6.8 μmol/L]; ALT/AST 341/271 U/L [ref 7–40 U/L/13–35 U/L]; ALP/GGT 302/213 U/L [ref 45–150 U/L (age-dependent)/7–45 U/L]; LDH 734 U/L [ref 120–250 U/L]; ADA 79.8 U/L [ref 0–20 U/L]; 5′-NT 34.2 U/L [ref 2–17 U/L]; prealbumin 91 mg/L↓ [ref 180–380 mg/L]; albumin 41.3 g/L↓ [ref 35–50 g/L]; total bile acids 229.8 μmol/L↑ [ref 0–10 μmol/L]. IgM 4.30 g/L↑ [ref 0.4–2.3 g/L]; PCT 0.258 ng/mL↑ [ref <0.05 ng/mL]. Direct antiglobulin test positive; indirect negative. CRP, coagulation profile, blood ammonia, and pancreatic enzymes were normal: CRP [ref <10 mg/L]; coagulation profile (typical refs: PT 11–14 s, INR 0.8–1.2, APTT 25–35 s, fibrinogen 2.0–4.0 g/L); blood ammonia [ref 15–45 μmol/L]; amylase 63 U/L [35–135 U/L] and lipase 34.2 U/L [13–60 U/L]; they were not re-measured later due to no clinical evidence of pancreatic injury. Ceruloplasmin and G6PD were within normal limits.

Imaging: Ultrasound (2025-09-24) showed gallbladder wall edema with lymphadenopathy around the porta hepatis and pancreatic head. CT (2025-09-25) displayed soft-tissue density from the hepatic hilum to the pancreatic head with mild intrahepatic ductal prominence and gallbladder high-density shadow (sludge-like stones to be excluded). MRI + MRCP (2025-09-29) revealed nodular signals adjacent to the portal vein and above the pancreatic head with mild lymphatic stasis; no intra-/extrahepatic ductal narrowing or dilatation; findings of cholecystitis less evident than on the 2025-09-25 CT.

Virology and hematology: EBV POCT positive (2025-09-26); EBV DNA 5.0 × 10^2^ copies/mL (reference <1.0 × 10^2^ copies/mL) on 2025-09-30. Tumor markers: CA 19-9 117.7 U/mL [ref <37 U/mL] (2025-09-25); CEA/AFP normal. Autoimmunity: ANA (titer <1:80), SMA, AMA-M2, anti-LKM-1, and other liver-related autoantibodies were negative (2025-09-28). Bone marrow aspiration smear (2025-10-03) showed active granulocytic/erythroid hyperplasia consistent with a reactive/stimulated marrow (no trephine/core biopsy).

### Diagnostic reasoning and clinical evolution

**Figure 2:**
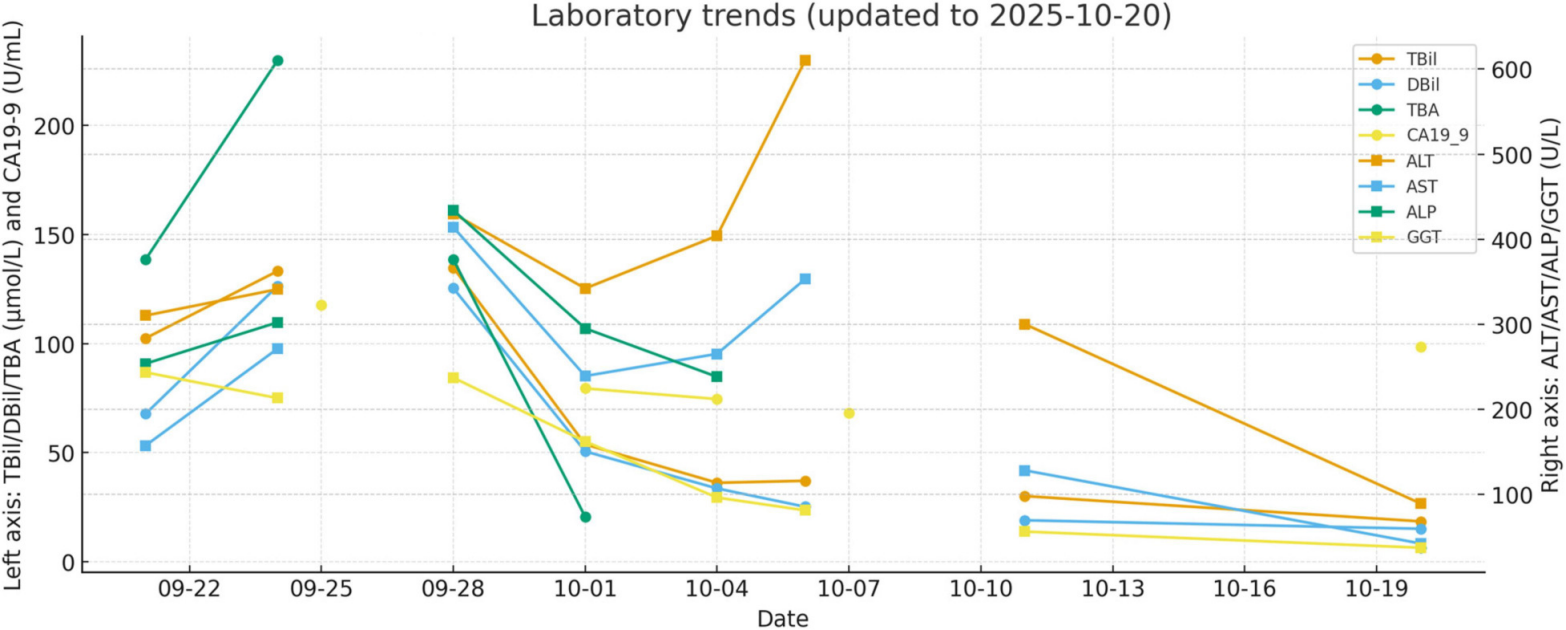
Laboratory trends (left axis: TBil/DBil/TBA/CA 19-9; right axis: ALT/AST/ALP/GGT).

For clarity, a structured clinical timeline correlating symptoms, laboratory trends, imaging findings, and clinical interpretation is provided in Figure 1 and Table 2. Key laboratory results (with reference ranges) are summarized in Table 1 and visualized in Figure 2.

**Table 1:** Key laboratory results (including follow-up to 2025-10-20).

Date	TBil (μmol/L; ref 3.4–20.5)	DBil (μmol/L; ref 0–6.8)	ALT (U/L; ref 7–40)	AST (U/L; ref 13–35)	ALP (U/L; ref 45–150; age-dependent)	GGT (U/L; ref 7–45)	TBA (μmol/L; ref 0–10)	CA 19-9 (U/mL; ref <37)
2025-09-21	102.4	67.7	310.1	157.1	253.5	243.3	138.5	—
2025-09-24	133.2	126.3	341.0	271.0	302.0	213.0	229.8	—
2025-09-25	—	—	—	—	—	—	—	117.7
2025-09-28	134.8	125.4	430.0	414.0	434.0	237.0	138.6	—
2025-10-01	53.7	50.5	342.0	239.0	295.0	162.0	20.5	79.4
2025-10-04	36.1	33.5	404.0	265.0	238.0	96.0	—	74.5
2025-10-06	37.0	25.1	610.0	353.0	—	81.0	—	—
2025-10-07	—	—	—	—	—	—	—	68.1
2025-10-11	30.0	18.9	300.0	128.0	—	56.0	—	—
2025-10-20	18.4	15.0	89.0	42.0	37.0	37.0	6.3	98.6

— denotes missing values. Units and reference ranges are indicated in the column headers.

**Table 2:** Structured clinical timeline with key laboratory trends, imaging findings, and clinical interpretation.

Date	Symptoms/PE	Key labs (ref in Table 1)	Imaging	Interpretation	Management
2025-09-18	Onset of jaundice and dark urine	—	—	Initial presentation	—
2025-09-21	Outside hospital evaluation	TBil/DBil 102.4/67.7; ALT/AST 310.1/157.1; ALP/GGT 253.5/243.3; TBA 138.5	US: small gallbladder; possible wall thickening	Cholestatic hepatitis; DAT+	Symptomatic therapy
2025-09-24	Admission; LAD; mild RUQ discomfort	TBil/DBil 133.2/126.3; ALT/AST 341/271; ALP/GGT 302/213; TBA 229.8	US: GB wall edema + porta hepatis/pancreatic head LAD	Cholestasis; broad differential	Conservative management; monitoring
2025-09-25	—	CA 19-9 117.7	CT: porta hepatis→pancreatic head density; mild ductal prominence	Consider obstruction vs. hepatocellular; malignancy assessed	—
2025-09-26	—	EBV POCT positive	—	EBV-IM favored	—
2025-09-29	—	—	MRI/MRCP: no fixed ductal stenosis/dilatation	Fixed obstruction unlikely at that time	UDCA started short off-label course
2025-09-30	—	EBV DNA 5.0 × 10^2 copies/mL	—	Low-level EBV DNA detected	—
2025-10-01	Improving symptoms	TBil/DBil 53.7/50.5; TBA 20.5; CA 19-9 79.4	—	Improving cholestasis and CA 19-9	Continue monitoring
2025-10-03	—	—	—	Bone marrow: reactive/stimulated	Hematologic malignancy unlikely
2025-10-04	—	TBil/DBil 36.1/33.5; CA 19-9 74.5	—	Continued improvement	UDCA completed (6 days)
2025-10-05	Discharge	—	—	Stable; improving	Outpatient follow-up
2025-10-20	Follow-up	TBil/DBil 18.4/15.0; ALT/AST 89/42; GGT 37; TBA 6.3; CA 19-9 98.6	US: unremarkable	Convalescence; mild CA 19-9 fluctuation	Trend-based monitoring

2025-09-21 (outside hospital): acute cholestatic hepatitis; limited response to symptomatic therapy (3, 17).

2025-09-24 (admission): differential included obstructive, hemolytic, infectious, metabolic, and immune causes, with significant cholestasis (TBil/DBil 133.2/126.3 μmol/L; TBA 229.8 μmol/L) (3, 4).

2025-09-25 (refinement): concern for obstructive vs. hepatocellular jaundice; CT hilum-to-pancreatic-head density; CA 19-9 117.7 U/mL [ref <37 U/mL] (CEA/AFP normal) (6, 16).

2025-09-26 (refinement): EBV-associated infectious mononucleosis (EBV-IM) favored (EBV POCT positive; ∼10% atypical lymphocytes; cervical/post-auricular lymphadenopathy) (4, 8, 11).

2025-09-27 to 2025-09-30: MRI + MRCP showed no intra-/extrahepatic ductal narrowing/dilatation; nodal changes were more consistent with inflammatory/reactive lymphadenopathy rather than a solid tumor causing fixed obstruction—evidence against mechanical obstruction (6, 7, 9). Given lymphadenopathy with atypical lymphocytes and CT abnormalities, hematologic malignancy (including pancreatic-head lymphoma) could not be fully excluded; bone marrow aspiration was therefore performed (17–19). A lymph node biopsy was not pursued because the abnormal nodes were deep (porta hepatis/peripancreatic) and not readily accessible without higher procedural risk, while superficial cervical nodes were clinically consistent with reactive lymphadenopathy. Given rapid clinical/biochemical improvement and a reactive marrow aspirate, a trephine/core marrow biopsy was deferred. The lack of histopathology is acknowledged as a limitation.

2025-10-01 to 2025-10-04: without endoscopic retrograde cholangiopancreatography (ERCP)/endoscopic ultrasound (EUS), TBil/DBil and TBA decreased markedly (53.7/50.5 to 36.1/33.5 μmol/L; TBA 20.5 μmol/L), CA 19-9 fell (117.7 to 79.4 to 74.5 U/mL), and ALP/GGT declined—consistent with reversible cholestasis-related CA 19-9 elevation. Bone marrow supported a reactive pattern, making hematologic malignancy unlikely (17, 18). Given negative MRCP for fixed obstruction, rapid lab improvement, and clinical stability without cholangitis, conservative management with close monitoring was favored over immediate ERCP/EUS (6, 7, 16). By 2025-10-05, the patient was discharged in improving condition.

## Therapeutic interventions

### Inpatient (2025-09-24 to 2025-10-04)

Therapeutic interventions (dose, route, and duration) are summarized in Table 3. Management prioritized etiologic evaluation, biliary imaging, and serial biochemical monitoring. Adjunctive medications were administered as short inpatient courses, with safety monitoring (serum electrolytes including potassium, blood pressure, and renal function).

**Table 3:** Therapeutic interventions and outpatient management.

Period	Therapy/management	Dose (per time)	Route	Frequency	Duration	Remarks
2025-09-24 to 2025-10-04	Compound glycyrrhizin (inpatient)	20 mL	ivgtt	qd	11 days	Adjunctive agent; safety monitoring
2025-09-24 to 2025-10-04	Glutathione (inpatient)	0.6 g	ivgtt	qd	11 days	Adjunctive antioxidant therapy
2025-09-24 to 2025-10-04	Ademetionine 1,4-butanedisulfonate (inpatient)	0.5–0.6 g	ivgtt	qd	11 days	Adjunctive therapy for cholestasis
2025-09-29 to 2025-10-04	Ursodeoxycholic acid	0.25 g	po	bid	6 days	Prescription-only; short off-label course after MRCP; discontinued
From 2025 to 10-05 (outpatient)	Glutathione tablets	0.2 g	po	tid	2–4 weeks	Adjust to labs/symptoms
From 2025 to 10-05 (outpatient)	Compound glycyrrhizin tablets	1 tablet	po	tid	2–4 weeks	Adjunctive agent; safety monitoring

Given initial concern for possible extrahepatic obstruction/compression on cross-sectional imaging, UDCA was withheld at presentation and was initiated only after MRI/MRCP on 2025-09-29 showed no obvious biliary obstruction. UDCA 0.25 g PO bid was then given for 6 days (2025-09-29 to 2025-10-04) as a short, prescription-only, off-label trial; the patient tolerated it without adverse events. We explicitly state that clinical recovery should not be interpreted as a causal treatment effect of UDCA or other medications.

Compound glycyrrhizin injection 20 mL, ivgtt, qd ×11 days (adjunctive agent; monitored electrolytes/BP/renal function).
Glutathione 0.6 g, ivgtt, qd ×11 days (adjunctive antioxidant therapy).
Ademetionine 1,4-butanedisulfonate 0.5–0.6 g, ivgtt, qd ×11 days (adjunctive therapy for cholestasis; no vitamin-B complex co-administered; tolerated).
Ursodeoxycholic acid 0.25 g, po, bid ×6 days (2025-09-29 to 2025-10-04; initiated after MRCP showed no obvious obstruction; off-label short course).

No antiviral therapy against EBV. No ERCP/EUS performed. Serum electrolytes (including potassium), blood pressure, and renal function were monitored; no adverse events were observed.

## Follow-up and outcomes

From 2025 to 10-01 onward, total/direct bilirubin and total bile acids declined markedly with parallel symptomatic improvement; the patient was discharged on 2025-10-05 with outpatient medications and follow-up.

2025-10-06: TBil/DBil 37.0/25.1 μmol/L; ALT/AST 610/353 U/L (transient peak); GGT 81 U/L; LDH 359 U/L. 2025-10-07: urine bilirubin 1+ (17 μmol/L), leukocyte esterase ± (15 cells/*μ*L), mucus threads 173/μL; stool routine normal. CA 19-9 68.1 U/mL. Superficial ultrasound: mildly enlarged bilateral cervical lymph nodes without fusion/liquefaction/calcification; axillary/inguinal nodes unremarkable. Abdominal ultrasound: no definite abnormal masses or ascites.

2025-10-11: TBil/DBil 30.0/18.9 μmol/L; ALT/AST 300/128 U/L; GGT 56 U/L [ref 7–45 U/L]; albumin 41.9 g/L [ref 35–50 g/L]. 2025-10-20: WBC 5.29 × 10⁹/L [ref 4.0–10.0 × 10⁹/L]; PLT 212 × 10⁹/L [ref 150–400 × 10⁹/L]; RBC 4.35 × 10^12^/L [ref 3.8–5.2 × 10^12^/L]; Hb 124 g/L [ref 120–160 g/L]; lymphocytes 32.9% [ref 20%–40%]; neutrophils 55% [ref 40%–75%]; CRP <0.80 mg/L [ref <10 mg/L]; no variant lymphocytes seen. Liver tests: TBil/DBil 18.4/15.0 μmol/L; ALT/AST 89/42 U/L; GGT 37 U/L; LDH 163 U/L; TBA 6.3 μmol/L. CA 19-9 98.6 U/mL. Abdominal ultrasound: hepatobiliary–pancreatic–splenic–renal scans unremarkable. Overall, bilirubin and liver enzymes continued improving with negative imaging; CA 19-9 showed short-term fluctuation at a low-to-moderate level during convalescence. We recommended re-measuring CA 19-9 in 2–4 weeks in the same laboratory and tracking TBil/DBil, ALP/GGT, and TBA to gauge residual cholestasis.

Discharge outpatient medications: glutathione 0.2 g po tid for 2–4 weeks; compound glycyrrhizin 1 tablet po tid for 2–4 weeks (adjust per liver biochemistry and symptoms). UDCA was discontinued after the 6-day inpatient trial and was not continued after discharge.

## Discussion

In adolescents, EBV-associated cholestatic jaundice with lymphadenopathy around the porta hepatis/pancreatic head and moderate CA 19-9 elevation can closely mimic malignant extrahepatic obstruction (9, 16, 19). Key elements supporting a benign, reversible process in this case were: (i) MRCP excluded fixed mechanical obstruction, thereby arguing against pancreatic head or cholangiocarcinoma (6, 7); (ii) bilirubin/TBA/ALP/GGT and CA 19-9 decreased in parallel during hospitalization, consistent with benign cholestasis dynamics (4, 5, 8); and (iii) EBV positivity with atypical lymphocytes and a reactive bone marrow pattern fits infectious mononucleosis spectrum; DAT positivity can occur in EBV infection with or without clinically significant hemolysis (11, 17, 18). Pancreatic-head lymphoma, although rare in adolescents, was considered in the differential diagnosis. In immunocompetent adolescents, EBV-related lymphadenopathy is usually self-limited; this case does not represent EBV-associated lymphoproliferative disease. DAT positivity in EBV infection may reflect transient autoantibody formation and can occur with or without clinically significant hemolysis; in this case, there was no progressive anemia or predominant indirect hyperbilirubinemia documented.

EBV-associated cholestatic hepatitis has been reported in both adults and children, including pediatric case series, and may present with an obstructive-appearing pattern on imaging due to inflammatory lymphadenopathy or gallbladder involvement (4, 20, 21, 26). Benign elevations of CA 19-9 have also been described in cholestasis and biliary inflammation; therefore, CA 19-9 should be interpreted in clinical context and alongside cholestatic markers rather than as a stand-alone marker of malignancy (22–24). Our case adds to the adolescent literature by illustrating the parallel decline of cholestatic markers and CA 19-9 with conservative management and a negative MRCP for fixed obstruction.

Other benign causes of CA 19-9 elevation include cholangitis and other biliary inflammation, as well as non-malignant hepatobiliary and systemic conditions. In this patient, the absence of clinical features suggestive of acute cholangitis (e.g., fever, hypotension, or worsening right upper-quadrant pain) and the rapid improvement in cholestatic indices argued against cholangitis as the primary driver. Pancreatic origin was unlikely given normal amylase/lipase. Drug- or exposure-related elevation was considered; no GLP-1 receptor agonists or over-the-counter phyto-oestrogen/herbal supplements were reported prior to presentation.

During outpatient follow-up, CA 19-9 fluctuated from 68.1 to 98.6 U/mL despite clinical/biochemical improvement. Potential explanations include post-inflammatory epithelial secretion/clearance lag, mild residual cholangiopathy, and analytic variability. In the absence of ductal dilatation or mass on imaging—and with falling bilirubin/GGT—malignancy is unlikely. Management should emphasize trend-based monitoring with repeat CA 19-9 in the same lab and concurrent TBil/DBil, ALP/GGT, and TBA; proceed to further imaging or invasive evaluation only if substantial, sustained rises occur with clinical or imaging red flags (5–14, 22, 24).

### Clinical implications of CA 19-9 testing

CA 19-9 was obtained because early cross-sectional imaging suggested a porta-hepatis/pancreatic-head lesion in the setting of cholestatic jaundice, making it reasonable to screen for pancreatobiliary malignancy despite its low pre-test probability in adolescents. However, CA 19-9 has limited specificity and may rise in benign cholestasis and inflammation; therefore, we suggest selective use (rather than routine testing) and trend-based interpretation alongside bilirubin and cholestatic enzymes, with further evaluation reserved for persistent or progressive elevation accompanied by clinical or imaging red flags (22, 24).

### Medication considerations

We avoided promotional language and do not recommend routine or preventive remedies. In suspected cholestasis with potential extrahepatic obstruction, etiologic evaluation and imaging localization are prioritized; UDCA was therefore withheld initially and used only briefly after MRCP did not show obvious obstruction. A STROBE-compliant study by Kotb MA et al. reported increased morbidity/mortality associated with UDCA use in infants with cholestasis; while this infant population differs from adolescents, it reinforces the need for caution and for avoiding general pediatric recommendations. Accordingly, we present UDCA here as a cautious, short-duration off-label course and do not infer efficacy from this course (25).

### Strengths and limitations

Strengths of this report include serial biochemical trends (bilirubin, ALP/GGT, total bile acids and CA 19-9) combined with CT/MRCP, supporting conservative management and avoidance of unnecessary invasive procedures. Limitations include the inability to fully exclude a transient extrinsic compression/partial obstruction that may have improved by the time MRCP was performed, and the lack of EUS/ERCP or histopathology to definitively characterize porta hepatis/pancreatic-head lymphadenopathy. Improvement should not be interpreted as evidence of efficacy of any single medication, and general recommendations cannot be made from a single case.

## Conclusion

EBV-related inflammatory lymphadenopathy can present in adolescents as cholestatic jaundice with transient CA 19-9 elevation, mimicking malignant obstruction. Negative MRCP for fixed ductal lesions and parallel improvement in cholestasis markers and CA 19-9 support a benign, reversible course; conservative management is often sufficient (6–8, 14).

## Patient perspective

As her mother, I was alarmed when I noticed my daughter's yellow skin and dark urine. When we learned that her CA 19-9 was elevated, we feared cancer. The clinicians explained that Epstein–Barr virus can cause cholestasis and that MRCP showed no biliary stricture, so we agreed to conservative management with close follow-up. Seeing her jaundice and liver tests improve over time was reassuring, and we appreciated an approach that minimized invasive procedures in an adolescent.

## Data Availability

The original contributions presented in the study are included in the article/Supplementary Material, further inquiries can be directed to the corresponding author.
